# Understanding the experience of healthcare providers in delivering care to older adults presenting in emergency departments: a qualitative study

**DOI:** 10.1186/s12913-026-15226-2

**Published:** 2026-07-30

**Authors:** Ling Zeng, Colleen Cheek, Lieke Richardson, Elizabeth Austin, Margaret Murphy, Matthew Vukasovic, Emilie Francis-Auton, Robyn Clay-Williams

**Affiliations:** 1 Australian Institute of Health Innovation, Macquarie University, 75 Talavera Road, North Ryde, Sydney, Australia; 2 Western Sydney Local Health District, Darcy Road, Westmead, Sydney Australia

**Keywords:** Emergency care, Healthcare provider, Older adult, Geriatric care, Health service, Health system

## Abstract

**Background:**

Knowledge gaps remain regarding the current challenges of delivering care to meet the needs of older adults presenting in the Emergency department (ED). This study aimed to explore the experiences of ED healthcare providers (HCPs) in providing care to older adults (OA).

**Methods:**

A qualitative descriptive approach was employed in this study, which was part of an ongoing five-year experience-based co-design project. Participants who were Australian registered clinicians (e.g. medical, nursing, and allied health), non-clinical staff who support clinical care (e.g., clerks and orderlies), or who manage the delivery of patient care were recruited from the EDs of three public hospitals in Sydney, Australia from March 2023 to April 2024. Semi-structured interviews and a focus group took place either in-person or via videoconference, depending on the participants’ preferences. Inductive thematic analysis was employed with no predetermined list of codes.

**Results:**

Forty-five semi-structured interviews and one focus group were conducted with 52 participants. A total of 34.6% participants were aged between 34 and 53 years old; 34 of 52 participants were female. Participants comprised 44% nurses, 27% doctors, and 17% allied health providers. Five major themes were identified: (1) patient complexity challenges care delivery, (2) organisational constraints challenge care delivery, (3) workforce issues undermine quality of care, (4) physical environments do not meet geriatric care needs, and (5) system interface issues constrain care transition. HCPs reported that excessive workload within time constraints, lack of resources, poor physical environment, and system interface issues create challenges in providing quality care to OAs with medical complexity. These challenges were further exacerbated by the mismatch between ED models of care and the needs of OAs.

**Conclusion:**

The development of a geriatric-specific ED pathway is likely to improve ED performance and better meet the needs of OAs. Partnering with ED HCPs and OAs offers a feasible approach to co-design a sustainable ED model of care and deliver quality of care that is geriatric-friendly.

**Supplementary Information:**

The online version contains supplementary material available at https://doi.org/10.1186/s12913-026-15226-2.

## Background

An aging population poses a growing challenge for all countries in terms of appropriate policy and funding to meet their healthcare needs. Globally, there will be approximately one in six people aged ≥ 60 years by 2030, and this number is projected to double (2.1 billion) by 2050 [1]. In Australia specifically, older adults (OAs) (≥ 65 years) comprised 16% of the total population, and one in eight OAs was aged 85 and over in 2020 [2]. The growing aging population contributes to a rising demand for Emergency Department (EDs) care worldwide [3, 4]. In 2023–2024, older Australians experienced a disproportionately high rate (23%) of ED presentations compared to the younger population (aged < 65 years), with 52% of them admitted to hospital following the ED presentation compared to 30% for all patients. People ≥ 85 years have the highest rate of presentation to ED for both males and females (921 presentations per 1,000 population for males and 778 per 1,000 population for females) [5]. Urgent change is needed if healthcare systems are to meet the needs of the rising number of OA who are increasingly presenting to EDs. In this study, old adults refer to those ≥ 65 years old.

The ED health care received by OAs is reported as suboptimal globally [6–9]. OAs are likely to have a higher risk of prolonged length of ED stay (≥ four hours) than the younger population, with the oldest old adults (≥ 85 years) at most risk of prolonged ED stay [10, 11]. Prolonged ED stay has been associated with increased risk of experiencing delirium, being sedated, adverse events (e.g., falls, pressure areas), in-hospital mortality and decline in physical function for OAs [8, 12–14]. The negative patient outcomes from prolonged ED stays can be partially attributed to the challenges in providing care to OAs with medical complexity, frailty syndromes and complicated social dynamics [9, 15]. It is likely that the suboptimal care received by OAs could be attributed to ED models of care not being well-designed for OAs [9, 16], where ED models of care do not account for evidence-based geriatric practice that supports the unique needs of OAs [17].

The development and implementation of new or adapted ED models of care to improve ED performance (e.g., reduced length of stay), improve patient outcomes (e.g. fewer adverse events) and improve patient experience (e.g. patient satisfaction) are warranted. It is essential to elicit the knowledge of ED healthcare providers (HCPs) to identify priority care gaps and involve ED providers in co-design. Existing literature has indicated that providing safe and high-quality care for older adults is often perceived as challenging by healthcare providers in ED [9, 15, 16, 18–21]. This has been commonly attributed to several factors, including insufficient geriatric knowledge and skills, the lack of integration between emergency care and community-based services, a mismatch between the traditional ED model of care and the complex and multifaceted needs of older patients, and the physical environment of EDs that is not well-suited to the care of geriatric populations [9, 15, 16, 18–21].

Previously studies, however, have predominantly explored the experiences of ED nurses in delivering care to OAs, rather than multidisciplinary perspectives in ED [18–21]. Knowledge gaps remain regarding the care challenges with ED practice that are current, Australian-specific, and consider a range of older adult needs, including those with advanced chronic disease and frailty who are being looked after in the community, or reside in aged care facilities receiving 24/7 care [9, 15, 16, 22]. This study aimed to comprehensively explore the experience of ED HCPs in providing care to OAs in three EDs in Australia.

## Methods

### Study design

This study was part of an ongoing five-year experience-based codesign ED project, which aimed to co-design and implement new or adapted models of ED care to improve ED performance, patient outcomes and patient experience for underserved patient cohorts [23]. In stage one of the overall ED project, a qualitative approach was employed to explore and understand the knowledge or service gaps ED HCPs perceived in providing ED care for four cohorts (older adults, adults with a cultural and linguistic background, adults with mental illness and people living with a disability). In this study, we report findings on ED HCPs’ experience in providing ED care to the OA cohort. The generated findings were used with those from a sample of older adult interviews to prioritise areas for codesign in stage two of the overall project. In accelerated codesign, quality improvement teams may use aggregated findings from reported literature to move more quickly to codesign [24], making comprehensive reporting of findings by researchers potentially valuable to others.

Compared to other qualitative approaches, qualitative description focuses on a rich understanding of the phenomenon, perspective or worldviews of the people involved, without going beyond the literal description [25, 26]. In line with the philosophical assumptions of naturalism and constructivism, this approach seeks to explore human phenomena or answer research questions through analysis and description of the meaning people ascribe to events in their natural context, and uses the generated knowledge to develop interventions, which is well-suited to health services research [25, 27]. The study was reported in line with the COREQ checklist.

### Study setting and sampling

The study sites were the EDs of three public hospitals within one local health district in metropolitan Sydney, Australia. Participants were Australian registered clinicians (e.g. medical, nursing, and allied health professionals), non-clinical staff who support clinical care (e.g., clerks and orderlies), or manage patient care delivery (patient experience coordinator), who had worked in one or more of the three nominated EDs for at least three of the previous 12 months. A pragmatic approach to participant recruitment was used to align with the clinical constraints of the ED setting and stated preferences of participants. The author team attended the ED to speak directly to ED staff at staff meetings about the study. On mutually agreed days, two author team members booked a room within the ED so that staff could drop in for short (up to 30 min) interviews. Alternatively, ED staff could select to participate in an interview or focus group via Zoom or Microsoft Teams at a different time with a research team member. At the beginning of interviews, participants were advised that they could terminate the interview at any time if they needed to attend to their clinical duties.

### Data collection

Prior to data collection, informed consent and initial demographic information were collected from eligible participants in Research Electronic Data Capture (REDCap), a secure web-based platform widely used for generating surveys and managing databases. Participants registered online or used an iPad at the time of the in-person interview. A single focus group was conducted with patient experience coordinators, who are non-health care professionals that provide an information service to patients and carers in the waiting room, and work across the three EDs. The semi-structured interviews and focus group took place predominantly in March – June 2023, with an additional two interviews collected in April 2024 from specialist geriatricians. All interviews and focus groups were audio-recorded. The same interview guide was used for both interviews and the focus group (see supplementary Table 1). Interviews and focus groups were conducted by four experienced qualitative researchers with PhD credentials (CC, LT, EA and RCW). Recruitment continued until data saturation was reached; that is, until no new information emerged from participants during the interviews [28].

### Data analysis

The audio files were transcribed verbatim using a professional transcription service and checked against the audio recording by members of the research team. During the process, all data were de-identified to ensure the participants’ confidentiality and finalised transcripts were imported into NVivo V.20 software for data analysis. Interview and focus group data were merged for data analysis.

Thematic analysis is a qualitative research method used to identify recurring patterns and underlying meanings within data, enabling researchers to generate rich descriptions and unanticipated insights [29]. Inductive thematic analysis was employed with no predetermined list of codes, following Braun and Clarke’s six-step guideline [29]. Consistent with the qualitative descriptive design in this study, semantic thematic analysis was conducted to produce the themes from the dataset that remained close to participants’ accounts and language, without excessive interpretation [29]. A qualitative analyst (LZ) familiarised herself with the data through reading the interview transcripts while undertaking de-identification, which also functioned as the initial stage of thematic analysis. The analyst inductively generated initial codes by reading the transcript line-by-line, coding data relevant to the research aims alongside continuously thinking: “Does this segment address the research aim? And if so, what meaning does it convey?”. These codes were subsequently examined, refined, and organised into preliminary themes and subthemes that represented shared patterns of meaning within the data. Two analysts (LZ and CC) then collaboratively reviewed and refined the themes and subthemes and their concomitant categories until consensus was reached. An example of the data coding process is presented in Table 1.

**Table 1:** An example of the coding process in data analysis

Data extraction	Codes	Sub-themes	Theme
*“Nowadays they come in*,* they’re on 20 to 30 different medicines. Because they’ve survived their six heart attacks and they’ve survived their years of hypertension and so forth. They’ve survived their kidney failure because of all this intervention.”*	Polypharmacy Multimorbidity	Medical complexity complicates clinical decision-making and practice	Patient complexity challenges care delivery
*“The elderly can be quite confused*,* we are not able to communicate as well. Those are the two areas that always cause a lot of concerns in terms of our clinical care.”* *“Someone (at the waiting room) will call you. Someone might have called*,* and they didn’t hear because their hearing wasn’t as good. Then they’ve been taken off the (ED waiting) list…*	Cognitive impairment Hearing impairment	Communication barriers compromise assessment and management	

In this study, data were collected from two sources: individual interviews and one focus group. Both data sources were analysed using the same thematic analysis process to identify recurring patterns and underlying meanings across the entire dataset. The coding generated from the focus group and individual interviews was compared, and demonstrated consistency in the emerging themes and subthemes across the two data resources. This supported their integration within a single analytical coding framework.

### Rigor and reflexivity

The research team are from diverse backgrounds, including allied health, nursing, engineering, sociology and psychology [30]. A description of the study participants, the location and the context of the research was elaborated to establish the transferability [30]. Researchers (CC, EA, LR, LT and RCW) who collected the data in the authorship team had no contact with any participants before the research and maintained a neutral and non-judgmental attitude toward participants. Prolonged engagement in data analysis was executed by LZ and CC, both registered nurses. Any discrepancies encountered during coding and analysis were discussed to achieve consensus. An audit trail was recorded for research-related decisions during the process. Findings from the analysis were checked by the authorship team, which includes practising ED HCPs.

## Results

### Characteristics of participants

Forty-five semi-structured interviews (mean duration: 26.5 min, ranging from 7 to 53 min) and one focus group (96 min) were conducted with a total of 52 participants. Six patient experience coordinators participated in the focus group. A total of 1289 min of interview data were analysed. Of the 46 transcripts, 41 highlighted experiences in providing care to OAs. There were 34.6% participants aged between 34 and 53 years old and 25% aged 54 and above; 34 of 52 participants were female. Participants comprised nurses (44%), doctors (27%), and allied health providers and others (17%), such as physiotherapist, occupational therapist, patient experience coordinator, and administrative staff who work in patient-facing areas. More than one-third had worked in ED for more than 15 years. Most participants had worked in one of the study EDs for more than 6 months of the previous year, and 92.3% spoke English at home (Table 2).

**Table 2:** The characteristics of participants (*N* = 52)

		Number	Percentage
Age groups (years)	*≤* 33	15	28.8%
	34–53	18	34.6%
	*≥* 54	13	25.0%
	No answer	6	11.5%
	Male	18	34.6%
	Female	34	65.4%
ED^1^ role	Nurse • Registered nurse • Advanced practice nurse • Nurse practitioner • Nurse unit manager • Other	23 8 9 1 3 2	44.2% 15.4% 17.3% 1.9% 5.8% 3.8%
	Doctor • Junior doctor • Registrar • FACEM^2^ • Other	14 2 2 6 4	26.9% 3.8% 3.8% 11.5% 7.7%
	Allied health or other	9	17.3%
	Patient experience coordinator	6	11.5%
Years worked in ED	0–2 3–6 7–10 11–14 15 or more	8 10 9 7 18	15.4% 19.2% 17.3% 13.5% 34.6%
Months worked in ED last 12 months	0–6 months 7–12 months No answer	5 46 1	9.6% 88.5% 1.9%
Speak English at home	Yes No	48 4	92.3% 7.7%

^1^ED: Emergency Department ^2^FACEM: Fellowship of National College Emergency Medicine

### The experiences of HCPs in delivering ED care to OAs

**Fig. 1:**
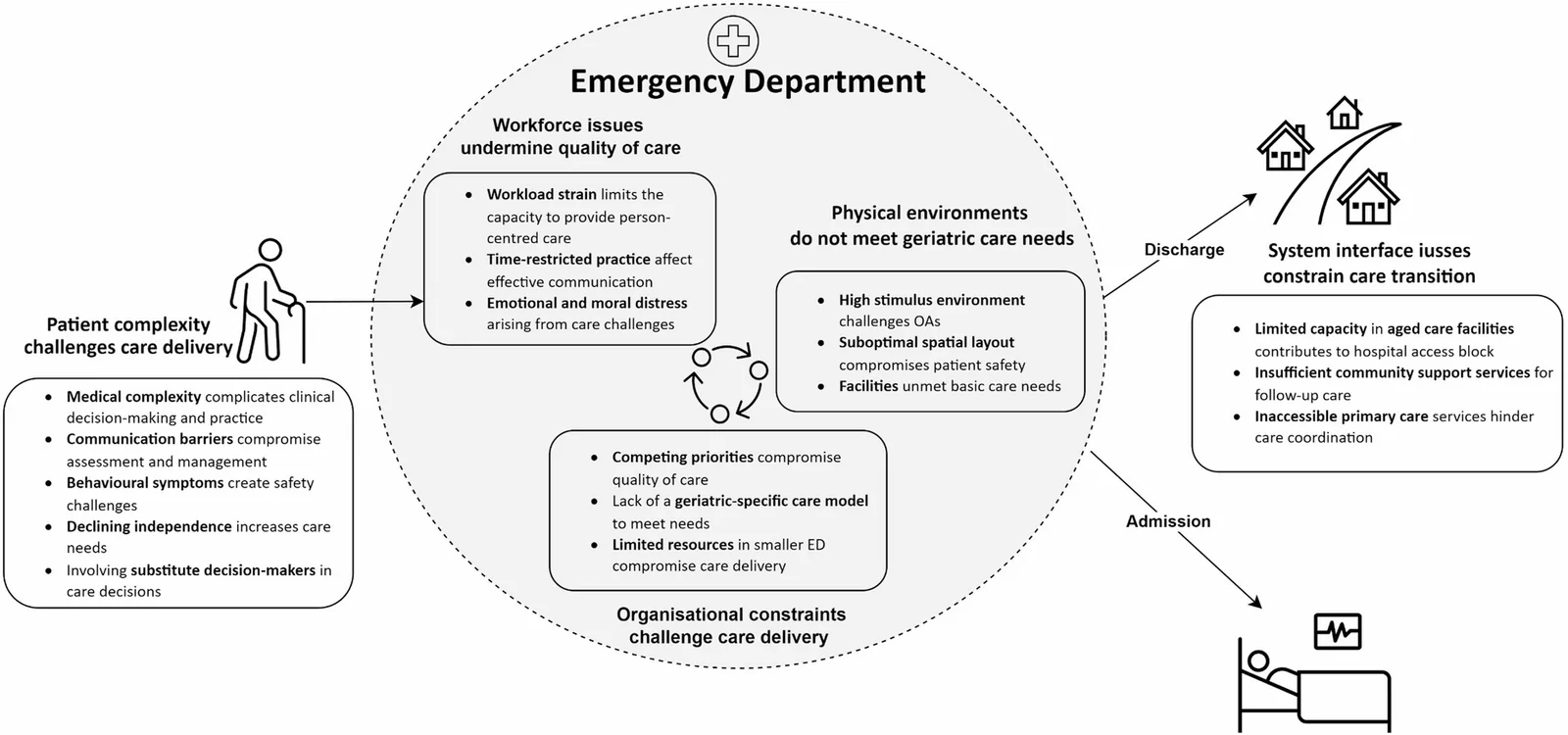
The experience of HCPs in delivering ED care to older adults

The experiences of HCPs in delivering ED care to OAs were organised into five major themes: (1) patient complexity challenges care delivery, (2) organisational constraints challenge care delivery, (3) workforce issues undermine quality of care, (4) physical environments do not meet geriatric care needs, and (5) system interface issues constrain care transition. Within each theme, subthemes specified each aspect of care delivery experience to OAs (see Fig. 1).

#### Theme one: Patient complexity challenges care delivery

##### Medical complexity complicates clinical decision-making and practice

ED Health Care Providers (HCPs) attributed the challenges in treating older adults (OAs) to the increased medical complexity among this cohort, involving multimorbidity, polypharmacy and risk of sudden deterioration. Multimorbidity made it difficult for ED HCPs to make a quick diagnosis and start timely interventions without more in-depth assessment and decision-making. The time involved in working through the OAs underlying multimorbidity was under-appreciated within the time and resource constraints of the ED. For OAs who were prescribed multiple medications, documenting an accurate and complete medication history was also time-consuming. OAs may miss their medications during the prolonged ED waiting or stay, which can contribute to further medical deterioration.

*So somebody may present with headache*,* but they’ve got diabetes and heart disease and other things and incidentally*,* we might find that their sugar is up or that their renal function is not working. So it’s not - it’s usually - in almost all cases*,* it’s not just a simple one system problem and with that goes polypharmacy. So they’ve got a whole list of medications and then when was the last time they had their medications checked?* (Specialist emergency physician 1)

##### Communication barriers compromise assessment and management

Many ED HCPs described communication barriers when interacting with OAs with cognitive impairment. It was challenging to gather medical information and conduct pain assessments on OAs who had dementia, delirium and Alzheimer’s disease, for example, as the individual was often unable to verbalise their needs or localise their pain. Hearing impairment was common in OAs and reported by ED HCPs as another barrier to communicating with them. The OAs, particularly those who did not bring their hearing aids to the ED, may miss their name being called in the waiting room. It was often time-consuming to obtain the required information from carers or aged care facility staff. The handover from some aged care facilities was unclear or sometimes lost detail due to staff shift changes. Some ED HCPs suggested that assessing patients’ communication limitations at initial assessment and adjusting communication style (e.g. repeat regularly and explain with patience and empathy) could facilitate communication.

*The elderly can be quite confused*,* we are not able to communicate as well. Those are the two areas that always cause a lot of concerns in terms of our clinical care*,* and we don’t meet every outcome that we’d want to meet as well.* (Registered nurse 1)

##### Behavioural symptoms create safety challenges

Managing aggressive behaviours and wandering or absconding behaviours were other challenges echoed by many ED HCPs in delivering care to OAs in the ED environment. For OAs with behavioural and psychological symptoms of dementia, agitation and aggression could be exacerbated by prolonged waiting and staying in the high-stimulus ED environment. The ED staffing ratios meant they could not provide the one-to-one care required to ensure the individual’s safety, resulting in a high risk of falls for these patients. OAs’ wandering behaviours could also be confronting for other patients and undermine others’ safety. ED HCPs valued the presence of a carer in the ED who had been taking care of an OA, for assisting with reassuring and settling the OA.

*So with the elderly*,* it’s a bit different but they get anxious. Especially when they’ve also got dementia plus mental health. It’s an issue for us because we are really short of staff and then these patients will have to wander around. We’re worried about the falls risk or she - he getting lost somewhere or absconding.* (Nurse unit manager 1)

##### Declining independence increases care needs

Functional decline among OAs was identified as a factor influencing care delivery. OAs with reduced independence required greater assistance with activities of daily living, including eating, toileting, mobility and pressure care, which increased care demands for HCPs. Given the fast-paced and time-constrained nature of the ED, many ED HCPs perceived the ED environment was not conducive to providing basic nursing care for OAs with declining independence and physical mobility.

*They’re all high falls risk. You know*,* the wards specialise in that sort of stuff. For us*,* we’re treating the immediate issue. We’re not as well trained as the wards to manage patients for long periods of time. Like where we need to order them air mattresses for their pressure injuries*,* we need to get them physio for their mobility.* (Nurse educator 1)

##### Involving substitute decision-makers in care decisions

Family members, as carers, advocates, and decision-makers, played crucial roles in supporting OAs in ED. Conflicts between family members on care decisions were sometimes challenging to manage.

*We had one yesterday evening in fact where we were calling the next of kin on file*,* couldn’t get in contact with the first one*,* called the second one*,* the second one they became irate between - and I then figured out that there was some kind of family rift between all of them*,* and it was like what have we started now? Because she’s*,* like*,* oh you called my brother*,* why did you call my – and it’s just - so*,* I’m not sure how you would do it….* (Specialist emergency physician 2)

#### Theme two: Organisational constraints challenge care delivery

##### Competing priorities compromise quality of care

Prolonged stay in ED meant basic care needs, such as assistance with Activities of Daily Living, pressure area care, and other longer-term considerations increased the scope of ED practice beyond initial assessment, stabilisation and referral. This basic nursing care was perceived by ED HCPs to be better provided in inpatient wards rather than ED, given the fast-paced nature of ED and the prioritisation of urgent acute care. It was also reported by some nursing staff the challenges in conducting over-engineered assessments and compliance documentation related to prolonged ED stay and having no time to attend to the personal care of OAs, which compromised the quality of care.

*We can do ED very well*,* it’s just when they can’t get to that longer stay*,* that’s where we start to really drop off in the level of care that we do give*,* because new things keep popping up. Higher priority tasks keep popping up and we somewhat neglect some of the basic cares that would get done if they were on – upstairs*,* or if they were in somewhere quieter.* (Nurse unit manager 2)

##### Lack of a geriatric-specific care model to meet needs

In some EDs, the lack of an on-site geriatric team and geriatric appropirate triage was reported by some ED HCPs to be a challenge in providing care to OAs. Lack of a multidisciplinary approach and effective communication involving the allied health team and specialist consultation, undermined the quality of care for OAs who experienced prolonged stays in the ED. ED HCPs strongly expressed a desire to develop and implement effective geriatric models of care in the ED for appropriate triage, early assessment and treatment along with close multidisciplinary collaboration.

*I think that it’s very important when they come to emergency department*,* that they should be triaged appropriately and receive the appropriate service*,* appropriate care with the appropriate service. If someone has a fall and they’ve got multiple rib fractures*,* well*,* they need to go under the trauma service. We have a geriatrician there*,* so they will be appropriately looked after.* (Geriatrician)

##### Limited resources in smaller ED compromise care delivery

Three areas of resource shortages were mentioned by ED HCPs, predominantly in the smaller ED, encompassing understaffing, lack of after-hours support and inadequate funding, which were detrimental to ED care delivery. Many ED HCPs expressed that understaffing resulted in low morale and stamina, which made them less time and were unable to provide person-centred care and guarantee the safety of care. HCPs raised concerns that limited after-hours support in the hospital, such as social services, pathology and pharmacist services on nights and weekends, delayed care delivery and prolonged OAs’ stay in ED. They attributed these limitations to inadequate funding for implementing multidisciplinary teams, specialist nurses, and pharmacists into ED care models.

*Busy*,* understaffed – like not appropriate nursing ratios*,* medical staff are overworked as well – we just have such a high presentation rate – numbers*,* and it’s just not matching up with our staffing – whether it be nursing and medical. So*,* yeah*,* patients just – they fall through the cracks*,* and they deteriorate*,* needing a resus bed.* (Nurse educator 2)

#### Theme three: Workforce issues undermine quality of care

##### Workload strain limits the capacity to provide person-centred care

Excessive workload under time constraints experienced by many ED HCPs was challenging, arising from increased ED presentations, medical complexity and behavioural issues among OAs. Prolonged ED stays increased the demand for supportive care, as older adults who experienced functional decline required assistance with basic activities of daily living. Understaffing and inadequate patient ratios further pushed ED HCPs’ limitations, which undermined the delivery of quality care to OAs.

*We try to help out as much as I can*,* but honestly speaking*,* a lot of the times because nurses are so drained out*,* that five minutes break you get*,* you’d rather just sit and be like let me just catch my breath. You don’t really think about helping somebody else. It’s something you can’t help it as well because you want to take care of yourself first.* (Registered nurse 2)

##### Time-restricted practice affects effective communication

A few ED HCPs stressed that poor patient interaction stemmed from communicating in medical language and a lack of time with OAs. HCPs strongly suggested that being an active listener, providing human comfort and showing empathy were features of comfort and therapeutic care that OAs value and assist in de-escalating agitation, if time was allowed. Notably, ED HCPs expressed the difficulties in initiating end-of-life conversations in time-pressured ED environments, which challenged aligning care with patients’ values.

*So when you’re having the first conversation (end-of-life plan) here about saying somebody*,* you know they’re not going to make it…but the families need to be ready to accept that*,* and that takes time. It’s not just you coming up*,* palliated you*,* family accept it*,* let’s move on. We can’t do that. It takes time. All those*,* they’ve very sensitive issues. Family need to come to terms with that.* (Geriatrician)

##### Emotional and moral distress arising from care challenges

ED HCPs reported that delivering care that did not meet the standard of their profession constituted a moral injury. Given the balancing of excessive workload within time-limited care environments, some nursing staff conveyed their frustration that they had no time to attend to the personal care of OAs, which failed to meet their own care standards. For some OAs with wandering or absconding behaviours, the impact of a fall could be catastrophic and further demoralising for ED HCPs.

*The hustle and bustle of an actual ED is that everything is like that [clicks fingers]*,* so you don’t have time very often*,* which is hard. As a nurse*,* you feel very unsatisfied with it. You don’t get to roll your patients and to check their skin and get the pressure mattress (Nurse educator 3)*

#### Theme four: Physical environments do not meet geriatric care needs

##### High stimulus environment challenges OAs

ED HCPs reported that the high stimulus environment, such as bright lights, noise, and a new and more chaotic physical environment than they experienced at home or residential facilities, was challenging for OAs. For OAs with cognitive decline, continuous artificial light coupled with lack of daylight in the ED, contributed to time-disorientation and sleep deprivation, which further triggered or exacerbated dementia and delirium. Patients became agitated with background noise that was irritating or interrupted their communication with ED HCPs. Notably, ED HCPs emphasised that a chaotic environment with mixed patients, new faces and a new physical environment was detrimental to OAs living with dementia, and contributed to prolonged stay in ED.

*Those that are in aged care facilities that have cognitive impairment*,* dementia and so on*,* all of the studies show that the longer you keep them out of the area that they’re used to in their aged care facility*,* bring them to a new area that’s quite busy*,* quite distracting*,* their deliriums*,* their acute deliriums*,* they get worse.*(Senior registered nurse)

##### Suboptimal spatial layout compromises patient safety

The ED physical environment was deemed unsafe by some ED HCPs for OAs, given the impact of some potential adverse events. Specific concerns included limited space and constricted layouts, which posed a high risk of injury from falls, particularly for OAs with behavioural symptoms. The exposure to some patients in the ED (e.g. mental illness, behavioural issues and prisoners) was not conducive to feelings of safety and security for OAs. On the other hand, the behavioural issues of some OAs could also be frightening to other ED patients. Thus, HCPs asserted that it would be beneficial to build a designated ED area for OAs to minimise adverse events (e.g. falls and aggression) and improve patients’ ED experience and safety.

*Then you’ve got all these other new faces and new people coming in*,* and all these noises*,* the telemetry. It’s not a very conducive atmosphere for them. It’s a high falls risk environment*,* because some of them*,* obviously*,* they get confused*,* they don’t know what to do*,* they just like to walk*,* and then it’s not favourable for them.* (Geriatrician)

##### Facilities unmet basic care needs

Some HCPs indicated that the ED physical environment was uncomfortable for OAs. This included, for example, cold air temperature, no food or drinks served in the waiting room except via a vending machine, uncomfortable chairs, and no privacy to discuss the treatment plan and progress with family or carers.

*So waiting in the waiting room*,* there’s no tea or coffee or lunch provided… they would ask for*,* yeah*,* you mainly need card for the vending machines…I don’t think we’ve ever had an elderly person ask for the vending machine.*(Clerk 2)

#### Theme five: System interface issues constrain care transition

##### Limited capacity in aged care facilities contributes to hospital access block

Some ED HCPs provided insight into the limited capacity of aged care facilities, in terms of bed availability and skills to manage behaviour and psychological symptoms, which contributed to challenges for OA flow through ED and hospital admission. OAs stuck in ED or hospital beds waiting for suitable aged care facility beds to become available resulted in prolonged stay in ED or hospital and contributed to hospital access block. Some ED HCPs felt aged care facilities sometimes referred OAs to ED for low acuity or trivial issues such as urinary catheter insertion and toe infection, and these “unnecessary presentations” increased the ED workload and exacerbated OAs’ cognitive decline. ED HCPs also appreciated that these “inappropriate ED presentations” resulted from a lack of nursing or treatment capacity in aged care facilities.

*But most of the geriatric patients*,* they come from the nursing home. They come for a very little and minimum thing that could have been sorted in the nursing home. They don’t need the ED presentation. So that’s unwanted load.* (Clerk 3)

##### Insufficient community support services for follow-up care

For some OAs, particularly those living alone, no access to a home care package or social services challenged ED HCPs to safely discharge them back to home, resulting in longer stays in ED and re-presentation to ED.

*There is a delay. Not so much from the community – like from health in itself*,* but just services at home*,* like cleaning or showers… So*,* that’s also a common thing that I’ve also noticed – that they’ve come back because nothing has started or can’t start because the services can’t start them.* (Occupational therapist)

##### Inaccessible primary care services hinder care coordination

A few ED HCPs perceived that ED presentations among OAs were related to the General Practice business model (a community fee-for-service system where care is predominantly funded through government funding, but sometimes a small gap payment is paid by the user). Others considered that while OAs may attend ED for low acuity care, their complexity and multi-morbidity were sometimes highly complex and therefore could not be managed in general practice.

*I might have mentioned to both of you before GP fees and the feasibility of GP businesses are jeopardised and so people are going well*,* it’s just simpler if I just come to the hospital. But if – do they have a GP and if they do*,* can they access the GP and does the GP have the skillset to manage that complexity?* (Nurse unit manager 3)

## Discussion

In this paper, we have comprehensively explored the experience of diverse ED HCPs in delivering care to OAs with advanced chronic illness or frailty. HCPs reported that excessive workload within time constraints, competing care priorities, lack of resources, poor physical environment, and system interface issues create challenges in providing quality care to OAs with medical complexity, cognitive impairment and declining independence. These challenges were further exacerbated by the mismatch between ED models of care and the particular needs of OAs with advanced illness or frailty. ED HCPs in this study strongly expressed the need to develop a geriatric-specific ED pathway to improve ED flow, reduce waiting time and adverse events, and better meet the needs of older adults.

Delivering care to OAs was challenging given their inherent advanced illness or frailty. This finding aligns with a previous study [31] conducted in Finland that found 85.7% of ED nurses and 96.9% of ED physicians reported feeling challenged to care for OAs. This situation can be further exacerbated when OAs present with behavioural issues. Our findings echoed previous research that more than half of HCPs reported having insufficient knowledge of geriatric care in ED [18, 31]. Numerous studies [9, 15–17] acknowledged the value of OAs’ carer involvement in ED care, particularly providing collateral information and assisting in mitigating behavioural escalation. HCPs in the current study emphasised the importance of developing a carer-friendly ED environment to help carers’ support of the OA.

There was a mismatch between the traditional acute ED model of care and the needs of OAs, which is consistent with previous studies [9, 15]. In 2023–2024, older Australians accounted for a high rate (23%) of ED presentations, with people aged ≥ 85 years having the highest rate of presentation to ED [5]. HCPs in our study indicated that caring for OAs required more time due to the greater complexity of their health conditions and treatment plans. The combination of higher ED utilisation and increased care complexity could lead to their longer ED stays. One recent study showed that the average length of stay at ED was 11.6 h among older adults (mean age 83.4) in Australia, and stays could stretch much longer (mean 14.7 h) for these OAs with admission needs [32]. Therefore, their longer ED stay extends into a period when they require assistance with activities of daily living and basic nursing care, which are not routine practices in the traditional acute ED model of care. Distress could rise among ED HCPs due to the tension between their professional and ethical obligation to provide the care that meets patient needs (including assistance in activities of daily living), and their stretched workload and insufficient geriatric-specific skills.

A geriatric-specific ED model of care that accounts for comorbidity by performing early assessment and treatment for OAs is a promising approach [16, 33]. However, these models must retain ED patient flow to support the primary function of the ED for critical emergency care, as well as minimise adverse effects and improve treatment outcomes [34]. Effective geriatric ED models of care also entail multidisciplinary collaboration, incorporating social workers, physiotherapists, pharmacists, and occupational therapists to meet the complex needs of older adults, particularly those still residing in the community. In addition, a geriatric-specific ED model of care is needed, regarding early identification of OAs who may benefit from palliative care approaches rather than escalated interventions.

Workforce constraints, such as excessive workload and time restrictions, were commonly reported by HCPs in the current study when delivering care to OAs. Specifically, the time and staffing required to attend to the basic nursing care needs of dependent older adults undermined the already stretched ED nurse-to-patient ratios, which have been previously identified across various qualitative studies [9, 15, 16]. HCPs in this study strongly suggested that assistants in nursing could be utilised to support basic nursing care or one-to-one specialist care, improve patients’ safety and minimise adverse events. Thus, increasing the EDs personnel capacity to meet basic care needs of OAs would be feasible to mitigate HCPs’ practice burnout and improve the quality of care to OAs.

In line with previous studies [9, 15], many ED HCPs in this study reinforced that the chaotic environment in ED was detrimental to OAs’ care, attributed to sudden cognitive or physical deterioration and amplification of their behavioural issues. Some perceived the traditional ED environment as a hazardous place of OAs, given the high fall risk and absconding behaviours. For example, the complex layout in ED with cluttered corridors and medical equipments, and unfamiliar surroundings with inadequate wayfinding signals (such as toilets), can create a hazardous environment for OAs, particularly for those with wandering and absconding issues, increasing the risk of falls and other adverse events. Moreover, sensory overload, such as the continuous 24-hour lighting in ED, can profoundly disrupt circadian rhythms and melatonin production of OAs living with dementia, which further undermines their sleep quality, exacerbates their cognitive impairment and prolongs overall recovery times [35]. Interventions or renovations in ED design to build a geriatric-friendly environment are urgently warranted. A geriatric-friendly and dementia-friendly environment promotes safety, independence and mobility, with day lighting, noise reduction, accessible navigation, window views to connect outside and non-slip floors proposed as favoured design features [36–38].

The current study is the first to explore the experience of delivering care to OAs from multiple disciplines within the ED system. However, there are some limitations to be considered when generalising our findings. Firstly, ED HCPs were recruited solely from one local health district with a comparatively low socioeconomic status in metropolitan Australia, the experiences of which may not apply in high socioeconomic or rural and remote areas. During the interview, participants’ sense of socially and professionally desirable responses may have generated data bias. Considering the differences between health systems and ED models of care nationally and internationally may constrain the relevance of the experiences we report. Data were collected from two sources: individual interviews and one focus group. Although consistency in the emerging themes and subthemes was found across the two data sources, the interactional dynamic inherent to the focus group may influence how participants express their perspectives. Although we noticed some interview lengths were short in that ED clinicians were called away for clinical duties, the collected data were sufficiently rich to generate comprehensive thematic findings that address the study aims. Finally, the transcripts were not provided to participants for feedback due to ED HCPs’ heavy clinical workload, which might have compromised study confirmability.

## Conclusion

HCPs in this study reported significant challenges in delivering high-quality care to older adults presenting in Emergency Department. These can be attributed to the medical and social complexity of geriatric patients, organisational constraints, workforce issues, the physical environment in ED being poorly matched to older adult needs, and system interface issues such as hospital access block affecting timely care transition. HCPs also offered recommendations to improve the delivery of emergency care to older adults. In particular, they proposed that development of a geriatric-specific ED pathway is urgently needed to better meet the needs of a growing older adult population and to improve ED flow, and reduce waiting time and adverse events. Partnering with ED HCPs and OAs offers a feasible approach to co-design an ED model of care and deliver geriatric-friendly quality of care for this population.

## Supplementary Information

Below is the link to the electronic supplementary material.

Supplementary Material 1

## Data Availability

The interview transcript data will be only accessed with participants’ permission, aligning with Human Research Ethics Committee approvals.
