# Supplementary material for: Understanding the experience of healthcare providers in delivering care to older adults presenting in emergency departments: a qualitative study
Source: BMC Health Serv Res. 2026 Jul 30;26:1490. doi: 10.1186/s12913-026-15226-2 (PMC13726201; doi:10.1186/s12913-026-15226-2)
Supplement: Supplementary file 1 [file 12913_2026_15226_MOESM1_ESM.docx]

**Supplementary Table 1: Interview guide_ ED healthcare providers**

*Please note that this guide only represents the main themes to be discussed with the participants and as such does not include the various prompts that may also be used (examples given for each question). Non-leading and general prompts will also be used, such as “Can you please tell me a little bit more about that?” and “What does that look like for you”.*

**Target cohorts**

(older adults, adults with a CALD background, adults with mental illness or adults with a disability)

Is there a specific cohort you would like to talk about today?

**1.Current care provision experience**

- Can you give us an example of when you were delivering care for these people in the ED?
- What went well?
- What were the challenges?

*Prompts:*  Constant lighting, noise, separation from significant others, cultural safety, Access block or overcrowding, organisation of care, 4-hour rule or other triage-based time-to-care targets, discharge/admission or transfer processes.

**2.Opportunities**

In your view, what is the main thing we must solve to improve care for this cohort?
